# PD-1-Targeted Discovery of Peptide Inhibitors by Virtual Screening, Molecular Dynamics Simulation, and Surface Plasmon Resonance

**DOI:** 10.3390/molecules24203784

**Published:** 2019-10-21

**Authors:** Yuanqiang Wang, Haiqiong Guo, Zhiwei Feng, Siyi Wang, Yuxuan Wang, Qingxiu He, Guangping Li, Weiwei Lin, Xiang-Qun Xie, Zhihua Lin

**Affiliations:** 1 School of Pharmacy and Bioengineering, Chongqing University of Technology, Chongqing 400054, China; guohq@2017.cqut.edu.cn (H.G.); 51181027109@2018.cqut.edu.cn (Q.H.); guangpingli@2019.cqut.edu.cn (G.L.); 2 Chongqing Key Laboratory of Medicinal Chemistry and Molecular Pharmacology, Chongqing 400054, China; 3 Chongqing Key Laboratory of Target Based Drug Screening and Effect Evaluation, Chongqing 400054, China; 4 State Key Laboratory of Silkworm Genome Biology, Southwest University, Chongqing 400715, China; 5 Department of Pharmaceutical Sciences and Computational Chemical Genomics Screening Center, School of Pharmacy, University of Pittsburgh, Pittsburgh, PA 15261, USA; zhf11@pitt.edu (Z.F.); siw29@pitt.edu (S.W.); wel83@pitt.edu (W.L.); 6 National Center of Excellence for Computational Drug Abuse Research, University of Pittsburgh, Pittsburgh, PA 15261, USA; 7 Drug Discovery Institute, University of Pittsburgh, Pittsburgh, PA 15261, USA; 8 Departments of Computational Biology and Structural Biology, School of Medicine, University of Pittsburgh, Pittsburgh, PA 15261, USA

**Keywords:** PD-1, peptides, molecular docking, molecular dynamics (MD) simulation, surface plasmon resonance (SPR)

## Abstract

The blockade of the programmed cell death protein 1/programmed cell death ligand 1 (PD-1/PD-L1) pathway plays a critical role in cancer immunotherapy by reducing the immune escape. Five monoclonal antibodies that antagonized PD-1/PD-L1 interaction have been approved by the Food and Drug Administration (FDA) and marketed as immunotherapy for cancer treatment. However, some weaknesses of antibodies, such as high cost, low stability, poor amenability for oral administration, and immunogenicity, should not be overlooked. To overcome these disadvantages, small-molecule inhibitors targeting PD-L1 were developed. In the present work, we applied in silico and in vitro approaches to develop short peptides targeting PD-1 as chemical probes for the inhibition of PD-1–PD-L1 interaction. We first predicted the potential binding pocket on PD-1/PD-L1 protein–protein interface (PPI). Sequentially, we carried out virtual screening against our in-house peptide library to identify potential ligands. WANG-003, WANG-004, and WANG-005, three of our in-house peptides, were predicted to bind to PD-1 with promising docking scores. Next, we conducted molecular docking and molecular dynamics (MD) simulation for the further analysis of interactions between our peptides and PD-1. Finally, we evaluated the affinity between peptides and PD-1 by surface plasmon resonance (SPR) binding technology. The present study provides a new perspective for the development of PD-1 inhibitors that disrupt PD-1–PD-L1 interactions. These promising peptides have the potential to be utilized as a novel chemical probe for further studies, as well as providing a foundation for further designs of potent small-molecule inhibitors targeting PD-1.

## 1. Introduction

Immunotherapy, a concept proposed by William B. Coley in 1891, is the treatment of diseases using the immune system [1]. This method was not applied to antineoplastic treatments until 1957 when Thomas and Burnet first established the theory of cancer immune surveillance. Cancer cells’ surface often contains tumor-associated antigens (TAAs) induced by genetic and epigenetic changes, which can be detected by the immune system. A variety of treatment methods have been developed over the years, such as adoptive T lymphocyte or T-cell therapy, anti-Cluster of Differentiation 47 (CD47) therapy, anti-Galleria Domain 2 (GD2) antibodies, immune checkpoints modulation, oncolytic virus, polysaccharides, and neoantigens. Among them, immunotherapy based on the regulation of immune checkpoints has harvested promising results in recent years. In the anticancer process, immune checkpoints are commonly up-regulated to inhibit the nascent anti-tumor immune response [2]. The inhibitors of programmed cell death protein 1 (PD-1), programmed death-ligand 1 (PD-L1), and cytotoxic T lymphocyte-associated antigen-4 (CTLA-4) have all been marketed as anti-neoplastic treatments (ipilimumab [3]; Bristol-Myers Squibb, CTLA-4 antibody; nivolumab [4], Bristol-Myers Squibb, PD-1 antibody; duvalumab [5], AstraZenaca, PD-L1 antibody).

PD-1, also known as CD279 [6], suppresses the inflammatory activity of T lymphocytes, which prevents autoimmune diseases through a series of apoptosis-related mechanisms: (1) Promotion of apoptosis of antigen-specific effector T cells (Teffs) that become tumor-infiltrating lymphocytes (TILs) at tumor sites; and (2) reduction of the apoptosis of regulatory T cells (Tregs), which plays the opposite role of Teffs by decreasing the proliferation of effector T cells. Once the tumor antigen is recognized, PD-L1 could be upregulated by interferon gamma (IFNγ), which is generated by TILs at the tumor site [7]. PD-1–PD-L1 interaction will protect the tumor from T-cell-mediated endogenous antitumor immune responses, which is called the immune escape. Therefore, the blockade of PD-1–PD-L1 interaction may promote the killing of cancer cells by reducing their immune evasion and could be the focus of the investigation in the immune checkpoint blockade (ICB) area [8].

Over the past decade, many drugs have been reported as PD-1/PD-L1 inhibitors. These drugs can antagonize PD-1–PD-L1 interaction and have been classified as antibodies (macromolecule protein) or small-molecule inhibitors [9]. The United States Food and Drug Administration (FDA) has approved five monoclonal antibodies that disrupt the PD-1–PD-L1 interaction (nivolumab [4], Bristol-Myers Squibb, 2014; pembrolizumab [10], Merck, 2014; atezolizumab [11], F. Hoffmann-La Roche AG, 2016; avelumab [12], Merck/Pfizer, 2017; and duvalumab [5], AstraZenaca, 2017). Nivolumab and pembrolizumab target PD-1, while atezolizumab, avelumab, and duvalumab target PD-L1. Approved indications vary among these antibodies. For example, nivolumab is recommended for the treatment of metastatic melanoma (MM), non-small cell lung cancer (NSCLC), metastatic renal cell carcinomas, advanced renal cell carcinoma, Hodgkin lymphoma, head and neck cancer, and urothelial carcinoma. Avelumab became the first treatment for patients with Merkel cell carcinoma to receive approval in the United States, Japan, and European Union [4]. Aside from these approved therapeutics, many other antibodies are in the process of clinical development; these antibodies include pidilizumab [13] (CureTech) that targets PD-1, along with MPDL3280A [14] (Genentech), MEDI4736 [15] (MedImmune, AstraZeneca), BMS-936559 [16] (Bristol-Myers Squibb), and MSB0010718C [17] (EMD Serono), which targets PD-L1. Effective antibody therapeutics have stepped into a renaissance era in recent years; however, the weaknesses of these antibody agents still exist. These limitations include high cost, low stability, poor amenability via oral administration, potential immunogenic side effects, etc.

To overcome these disadvantages, small-molecule inhibitors were developed. Bristol-Myers-Squibb (BMS) has designed and patented a serial of compounds, including BMS-1001 and BMS-1166, which bind to human PD-L1 to block its interaction with PD-1 [8]. Recently, Guzik et al. [18] reported some small-molecule inhibitors of PD1/PD-L1, which target PD-L1. Han et al. [19] screened caffeoylquinic acid compounds by surface plasmon resonance (SPR), which showed the potential to bind to PD-1 at the level of nanomoles to target the PD-1/PD-L1 pathway. Similarly, Donnelly et al. [20] used ^18^F-labeled BMS-986192, a compound with a lower dissociation constant (<35 pM), to image PD-L1 expression. These works raised the hope of small-molecule inhibitors as candidates for the disruption of PD-1–PD-L1 interaction. However, small-molecule inhibitors still face many challenges due to the incomplete knowledge of the PD-1/PD-L1 anticancer pathway and the relatively flat and highly hydrophobic PD-1–PD-L1 interaction surface [21]. Fortunately, low molecular weight synthetic peptides have the same advantages as small-molecule inhibitors, including lower manufacturing costs, higher stability, reduced immunogenicity, and better organ and tumor penetration compared to anti-body therapeutics [22]. Additionally, synthetic peptides are larger compared to small-molecule agents, which may result in stronger interaction when interfering with the interface of PD-1–PD-L1 by creating multiple hydrogen bonds.

In the present work, we reported short peptides (WANG-003, WANG-004, and WANG-005) that target PD-1 to disrupt the PD-1–PD-L1 interaction, which shows a new perspective for developing inhibitors of PD-1/PD-L1 and provides novel chemical probes for related studies.

## 2. Materials and Methods

### 2.1. Reagents and Materials

Series S sensor chip CM5, HBS-EP buffer (10×, 0.1 M HEPES (4-(2-hydroxyethyl)-1-piperazineethanesulfonic acid) buffer with1.5 mM NaCl, 30 mM EDTA (Ethylenediaminetetraacetic acid), and 0.05% surfactant P20, pH 7.4), Amine Coupling Kit [1-Ethyl-3-(3-dimethylaminopropyl)-carbodiimide hydrochloride (EDC), *N*-Hydroxysuccinimide (NHS) and 1.0 M ethanolamine-HCl (pH 8.5)], immobilization buffer (sodium acetate pH 4.5), and regeneration solutions (NaOH 50 mM; 10 mM glycine 2.5; 10 mM glycine 3.0, for analysis) were purchased from GE Healthcare Life Science (Uppsala, Sweden).

### 2.2. Synthetic Peptides and Proteins

In the reaction column, 20 mL of DCM (Dichloromethane or methylene chloride) were added to a commercially available 2-chloride resin, (0.13 g, loading 0.8 mmol/g), and then oscillated for 30 min to activate the resin. Additionally, 1.05 equivalents of l-Trp (BOC)-OH and 10 equivalents of DIEA (diisopropylethylamine) were added. Otherwise, a small amount of DMF (Dimethylformamide) was solubilized. After gently stirring the mixture for 1 h, the resin was washed with DMF and DCM. The first amino acid was deprotected by 20 mL of 20% piperidine/DMF, then oscillated for 20 min. The coupling was confirmed via a chloranil test [23]. The coupling was followed by washing of the resin with DMF (3 × 5 min) and methanol (2 × 5 mL). Coupling was undertaken using a pre-activated solution of Fmoc-l-Arg(Pbf)-OH (3 molar equivalents), HBTU (*O*-(Benzotriazol-1-yl)-*N*,*N*,*N*′,*N*′-tetramethyluronium hexafluorophosphate, 3 molar equivalents), and DIEA (10 molar equivalents), followed by incubation for 30 min. The second washing was repeated. The cycles of deprotection and coupling were repeated until the desired peptide chain length was obtained. The resin-bound peptide was transferred to a round-bottomed flask, and the simultaneous removal of resin and the protective groups was achieved by using a cocktail combination of TFA:H_2_O:EDT:TIS (94.5:2:2.5:1) for 2 h. The crude peptide was filtered and purified on a preparative HPLC system and analyzed using the mobile phase of acetonitrile (95%)/H_2_O (5%) in the gradient system: 30 min gradient, acetonitrile (95%)/H_2_O (5%) at 214 nm [24].

Peptides of lyophilized powder were dissolved in sterile milli-Q H_2_O to a stock concentration of 1 mM for use in surface plasmon resonance (SPR) analysis. Recombinant human PD-1 protein and recombinant human PD-L1 protein were purchased from Abcam (Cambridge, UK) and reconstituted in the sterile running buffer according to the manufacturer’s protocol to a stock concentration of 1 mg/mL and 400 ug/mL, respectively. All synthetic peptides and proteins were equally packaged to avoid repeated freeze–thaw cycles.

### 2.3. Human PD-1 3D Structure Preparation

In the present work, the crystal structure (PDB ID: 4ZQK) [25] and protein sequence (Uniprot ID: Q15116) of PD-1 were obtained from the Protein Data Bank (PDB, www.rcsb.org) and Uniprot (www.uniprot.org) databases, respectively. Residual repairs and energy minimization were then carried out using SYBYL.

### 2.4. Virtual Screening and Molecular Docking

According to the hydrophobic interface of the PD-1–PD-L1 complex, the binding pocket was defined based on the key residues on PD-1 that contributed to their interactions. These residues include Val64, Tyr68, Gln75, Thr76, Lys78, Asp85, Ile126, Leu128, Ala132, Ile134, and Glu136. The docking program Surflex-Dock GeomX (SFXC) in SYBYL-X 1.3 was utilized to carry out the virtual screening and construction of PD-1-peptide complex in which the docking scores are expressed in −log_10_ (K_d_) [26]. The main protocols or parameters of docking are listed as follows [27,28,29,30,31]: (a) The “number of starting conformations per ligand” was set to 10, and the “number of max conformations per fragment” was set to 20; (b) the “maximum number of rotatable bonds per molecule” was set to 100; (c) flags were turned on for “pre-dock minimization”, “post-dock minimization”, “molecule fragmentation”, and “soft grid treatment”; (d) “activate spin alignment method with density of search” was set to 9.0; and (e) the “number of spins per alignment” was set to 12.

Briefly, the exact protocol employed for the hits’ selection during virtual screening was as follows: (a) First, 10 starting conformations for each in-house peptide were generated; (b) each peptide was then docked to the potential pocket in PD-1 using the docking algorithm implemented in SYBYL and the top 20 binding poses of each peptide with higher docking scores were saved; (c) the best docking score of each peptide was used for the sorting; and (d) the top three compounds with the highest docking scores were selected as the hit compounds for further analyses.

### 2.5. Molecular Dynamics (MD) Simulation

Two systems were set up by leap and included 50,489 water molecules, 137 Na^+^ ions, and 140 Cl^−^ ions. The initial conformation of the complex was taken from docking studies. The sizes of the initial simulation boxes were ~ 122 Å ×122 Å × 122 Å. The AMBER ff14SB force field [32] was applied to proteins and the AMBER Lipid14 force field [33] was applied to lipids. Water molecules were treated with the TIP3P water model [34].

The MD simulations were carried out using the PMEMD.mpi and PMEMD.cuda modules in the AMBER16 [35,36,37] package. First, several minimization steps were carried out for the systems to avoid possible steric crashes. Then, each system was gradually heated from 0 to 300 K during the heating stage and kept at 300 K during the following equilibrium and production stages. A time step of 2 fs was used for the heating stage, equilibrium stage, density adjustment stage, and the entire production stage. A periodic boundary condition was employed to maintain constant temperature and pressure (NPT) ensembles. The pressure was set at 1 atm and controlled by the anisotropic (x^−^, y^−^, z^−^) pressure scaling protocol, with a pressure relaxation time of 1 ps. The temperature was regulated using Langevin dynamics with a collision frequency of 2 ps^−1^ [38,39]. The particle mesh Ewald (PME) method [40,41] was adopted to handle long-range electrostatics and a 10 Å cutoff was set to treat real-space interactions. All covalent bonds involving hydrogen atoms were constrained with the SHAKE algorithm [42]. Each system was carried out with 200 ns MD simulation and the trajectory of simulated systems was saved every 100 ps.

### 2.6. Molecular Mechanics/Generalized Born Surface Area (MM/GBSA) Calculation

For the saved trajectories of MD simulations, the molecular mechanics/generalized born surface area (MM/GBSA) [43,44,45] method was used to calculate the binding energies of receptors under the treatment of different ligands, respectively. In total, 150 snapshots were extracted from each trajectory every 200 ps from 170 to 200 ns in order to calculate the mean binding energy. The following formula was used:

△*E*_bind_ = △*E*_MM_ + △*E*_SOL_ = △*E*_MM_ + △*E*_GB_ + △*E*_SA_ (1)

where △*E*_bind_ is the binding energy and △*E*_MM_ denotes the sum of molecular mechanical energies in vacuo and can be further divided into the contributions from electrostatic, van der Waals, and internal energies. This term could be computed through a molecular mechanics method. △*E*_SOL_ is the solvation energy, which includes the polar solvation energy (△*E*_GB_) calculated with the generalized born (GB) approximation model [46,47], and the non-polar part (△*E*_SA_) obtained by fitting the solvent accessible surface area (SASA) [48] with the LCPO (Linear Combination of Pairwise Overlaps) model [49,50]. Additionally, the energies of each residue were decomposed into backbone and side-chain atoms. Analysis of key residues’ energy contributions during binding was made possible through energy decomposition [51].

### 2.7. Surface Plasmon Resonance (SPR) Analysis

The affinities constant (KD) and kinetics (ka and kd) of peptide immunomodulator or PD-L1 binding to PD-1 were assayed using Biacore X100 (GE Healthcare, Sweden), all at 25 °C. The HBS-EP buffer (10×) was diluted 10-fold with sterile deionized water, filtered through a 0.22-μm membrane filter, and degassed immediately before the experiment as running buffer with a flow rate of 30 μL/min for the instrument system. The purified active PD-1 was diluted by 10 mM sodium acetate solution at pH 4.5, resulting in a protein concentration of 50 μg/mL. Coupling conditions were determined by protein isoelectric points and previous reports [52]. The diluted protein was immobilized on the surface of a CM5 sensor chip via the primary amine group, employing a standard Amine Coupling Kit, and the target immobilization level was 5000 response units (RUs). The flow cell 1 with any modification was used as a reference to correct the system error. The RU values were collected, and all the binding affinity data was calculated by kinetic models (1:1 interaction) within Biacore X100 Evaluation Software.

To determine the binding affinity between PD-1 and PD-L1, a series of protein dilutions was analyzed by multi-cycle kinetics/affinity. A concentration gradient of PD-L1 as the analyte was freshly prepared in HBS-EP running buffer with at least five concentrations. The PD-L1 at various gradient concentrations (including a repeat concentration) and one zero concentration (running buffer) flowed over the immobilized PD-1, with 120 s for binding, followed by disassociation for 120 s, and the obtained response units (RUs) were recorded. After the injection of every concentration of PD-L1, the sensor chip surface was regenerated with 10 mM glycine 2.5 for 30 s to completely remove the residual PD-L1.

The designed peptide was dissolved and diluted with HBS-EP buffer to in a series of concentrations (2–64 μM for WANG-003, 1–16 μM for WANG-004, 1.5625–100 μM for WANG-005, 62.5–1000 μM for WANG006, and 3.125–100 μM for WANG007). Each concentration was injected into the channel for 120 and 150 s for dissociation. Then, 10 mM glycine 3.0 or glycine 2.5 was utilized to regenerate the sensor chip surface. The equilibrium dissociation constant (KD) was calculated to evaluate the ability of peptides to interact with PD-1.

## 3. Results and Discussion

### 3.1. Potential Binding Site on PD-1 Protein Interface

**Figure 1:**
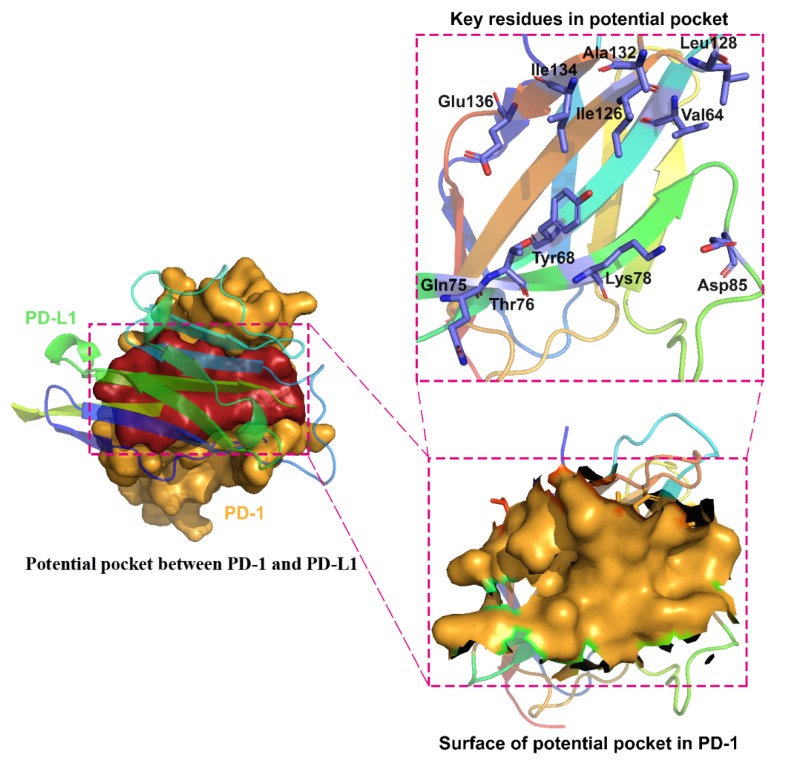
The potential binding site between PD-1 and PD-L1. Key residues represented in blue in the potential pocket include Val64, Tyr68, Gln75, Thr76, Lys78, Asp85, Ile126, Leu128, Ala132, Ile134, and Glu136. The surfaces of the potential pocket in PD-1 are presented in yellow.

Several tools were utilized for the characterization of the PD-1 interface, including CASTp [53], MAPPIs [54], MultiBind [54], MolSurfer [55], and ProFace [56], to ensure the consistency of the prediction. One potential binding site on the protein was found by all tools utilized, as shown in Figure 1. The binding pocket was formed by PD-1 residues, including Val64, Tyr68, Gln75, Thr76, Lys78, Asp85, Ile126, Leu128, Ala132, Ile134, and Glu136. All of the listed residues have been reported to contribute to the binding between PD-1 and PD-L1 [25].

### 3.2. In-House Peptides as PD-1 Inhibitors/Modulator

In total, 30 in-house peptides were synthesized previously as chemical probes for anticancer, antiviral, and antimicrobial agents. In the present work, virtual screening against our in-house peptide library was carried out to identify potential PD-1 inhibitor(s)/modulator(s), using the refined PD-1′s 3D model. Three peptides with the highest docking scores were selected as the potential hits and underwent further experimental validation.

From the results of the HPLC (high performance liquid chromatography) (Table 1, Supplementary Information Figures S1–S4), the purity of our inhouse peptides was all higher than 95%. Furthermore, the molecular weights of the synthetized peptides obtained from MS (relative molecular mass) analysis were consistent with the theoretical calculation (Table 1, Supplementary Information Figures S1–S4).

### 3.3. Detailed Interactions and MD Simulation of PD-1/Peptides

#### 3.3.1. Interactions between PD-1 and WANG-003

**Figure 2:**
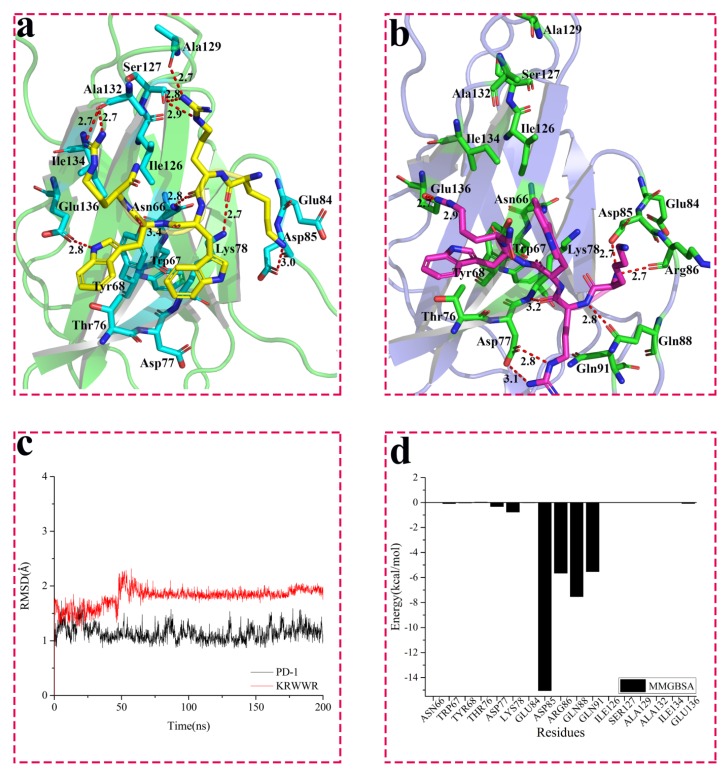
Interaction between WANG-003 (KRWWR) and PD-1. (**a**) Interaction between WANG-003 (KRWWR) and PD-1; (**b**) Interaction between WANG-003 (KRWWR) and PD-1 after 200 ns MD simulation; The root mean square deviation (RMSD) for the backbone (@C, CA, N, O) of PD-1 (**c**) and Molecular Mechanics/Generalized Born Surface Area (MM/GBSA) (**d**) of PD-1 with inhibitor for 200 ns (MD) simulation.

As described in the previous protocol, WANG-003 (KRWWR) was docked into the crystal structure of PD-1 protein. As shown in Figure 2a, important residues involved in the binding pocket included Asn66, Tyr68, Lys78, Asp85, Ser127, Ala129, Ala132, and Glu136. All residues described here were consistent with the information acquired from the potential PPI binding site between PD-1 and PD-L1. According to the docking results, Ser127 (2.8 and 2.9 Å) and Ala129 (2.7 Å) interacted with WANG-003 via hydrogen bonds, and two additional hydrogen bonds (2.7 Å) were formed between Ala132 and the guanidyl of Arg5 on the peptide. Importantly, Asp85 and Glu136 played a role in the hydrogen bonding interaction of PD-1 with WANG-003.

To explore the dynamic interactions among PD-1 and peptides, and investigate the role of the contacted residues, 200 ns MD simulation of the complex of PD-1/peptides was performed. For the system of PD-1/WANG-003, the root mean square deviation (RMSD) plot (Figure 2c, black and red line) shows that both PD-1 and WANG-003 remained stable after 60 ns MD simulation, with fluctuation around 2 Å. Figure 2 compares the conformational change of WANG-003 and PD-1 between pre-MD and post-MD. After 200 ns MD simulation, the complex of WANG-003 and PD-1 became more stable than its docked pose. Moreover, the conformation of WANG-003 shifted slightly for a more favorable pose during the MD simulation, forming a stronger interaction with PD-1: (1) Asp85 and Arg86 in PD-1 formed two hydrogen bonds with Lys1 of WANG-003, with a distance of 2.7 Å, and the carbonyl of Gln88 on PD-1 also had a hydrogen bond with a distance of 2.8 Å with acyl of Lys1; (2) the carboxyl of Asp77 on PD-1 formed strong hydrogen bonds (2.8 and 3.1 Å) with the guanidyl of Arg2 on WANG-003, and a hydrogen bond (3.2 Å) also existed between the acyl of Lys78 and the carbonyl of Arg2; and (3) moreover, the carboxyl of Glu136 on PD-1 formed two intense hydrogen bonds (2.7 and 2.9 Å) with the guanidyl of Arg6 on WANG-003.

To explore the contribution of contacted residues for the binding of WANG-003, the free energy was decomposed using the MM/GBSA method, as shown in Figure 2d and Table S1 in the Supplementary Information. Analysis was focused on several key residues, including Asp77, Lys78, Asp85, Arg86, Gln88, and Glu91. The results showed that these key residues contributed to a negative total energy for the binding of WANG-003. They contributed larger free energy through electrostatic interactions due to the formation of seven strong hydrogen bonds with WANG-003 (Table S1). The free energy contribution was consistent with the binding mode of PD-1/WANG-003 and provided more details for their interaction.

**Figure 3:**
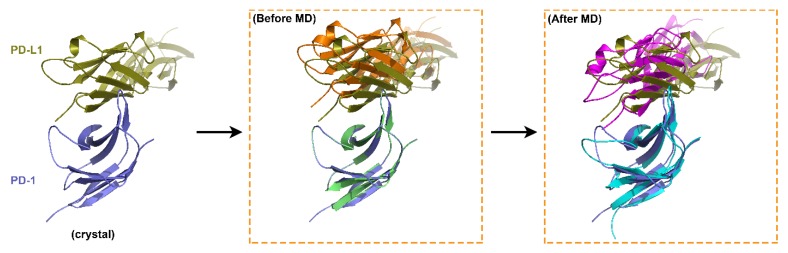
The binding mode of PD-1/PD-L1 after MD. WANG-003 was removed from the system of PD-1/WANG-003/PD-L1 to construct the complex of PD-1/PD-L1 and 100-ns MD simulation was performed. After the MD simulation, the constructed PD-1/PD-L1 was aligned with the crystal structure and the two systems were found to be superimposable.

For comparison, WANG-003 was removed from the system of PD-1/WANG-003/PD-L1 to construct the complex of PD-1/PD-L1 and another MD simulation was performed. The only difference between the in-house-constructed PD-1/PD-L1 and the crystal structure was that the molded PD-L1 was slightly further away from the molded PD-1 (Figure 3, middle, orange). After the MD simulation, the in-house-constructed PD-1/PD-L1 was aligned with the crystal structure and the two systems were superimposable (Figure 3, right, purple). Taken together, our results showed that WANG-003 has the potential to block interactions between PD-1 and PD-L1.

#### 3.3.2. Interactions between PD-1 and WANG-004

**Figure 4:**
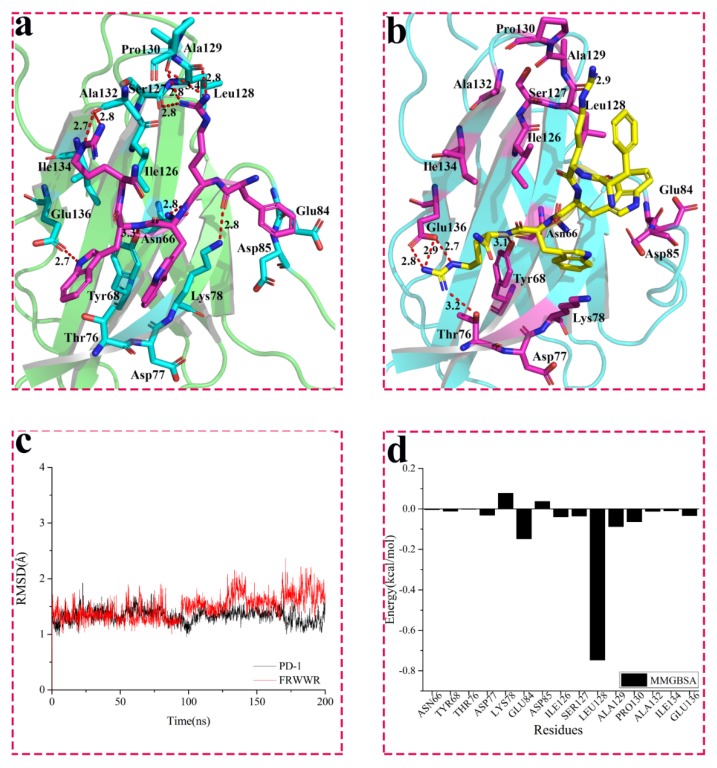
Interaction between WANG-004 (FRWWR) and PD-1. (**a**) Interaction between WANG-004 (FRWWR) and PD-1; (**b**) Interaction between WANG-004 (FRWWR) and PD-1 after 200 ns MD simulation; The root mean square deviation (RMSD) for backbone (@C, CA, N, O) of PD-1 (**c**) and Molecular Mechanics/Generalized Born Surface Area (MM/GBSA) (**d**) of PD-1 with FRWWR for 200 ns molecular dynamics (MD) simulation.

Furthermore, WANG-004 (FRWWR) was also docked into PD-1 to study their interactions. The binding mode of WANG-004 with PD-1 is shown in Figure 4. In the docking results, strong hydrogen bonds were observed between key residues, including Asn66 (2.8 Å), Tyr68 (3.3 Å), Lys78 (2.8 Å), Ser127 (2.8 Å), Leu128 (2.8 Å), Pro130 (2.8 and 3.4 Å), Ala132 (2.7 and 2.8 Å), Glu136 (2.7 Å), and WANG-004.

Then, we explored the interactions between PD-1 and WANG-004 by carrying out a 200 ns MD simulation for this complex. The RMSD plot of PD-1/WANG-004 fluctuated around 1.3 and 1.5 Å, respectively (black and red lines in Figure 4c). Meanwhile, the average structure of the last 30-ns simulations was generated and used for analyses. Our results showed that 200 ns MD simulation for the complex of PD-1 and WANG-004 resulted in some conformational changes (Figure 4). The carbonyl of Leu128 on PD-1 formed a hydrogen bond (2.9 Å) with the guanidyl of Arg2 on WANG-004; the hydroxyl of Tyr68 on PD-1 had hydrogen bonding (3.1 Å) with the acyl of Trp4 on the peptide. It is noteworthy that the guanidyl of Arg5 formed strong hydrogen bonds with both the carboxyl of Glu136 (2.7, 2.8, and 2.9 Å) and the hydroxyl of Thr76 (3.2 Å) on PD-1.

The MM/GBSA method was performed to investigate the contribution of contacted residues for the binding of WANG-004. Figure 4d and Table S2 show that these key residues, including Glu84, Ile126, Leu128, Ala129, Pro130, and Glu136, contributed a negative total energy for the interaction of PD-1 and WANG-004. Furthermore, the contribution of the carbonyl of Leu128 was the dominating factor for the binding. All these MD results provide detailed information on the interactions between PD-1 and WANG-004.

#### 3.3.3. Interactions between PD-1 and WANG-005

**Figure 5:**
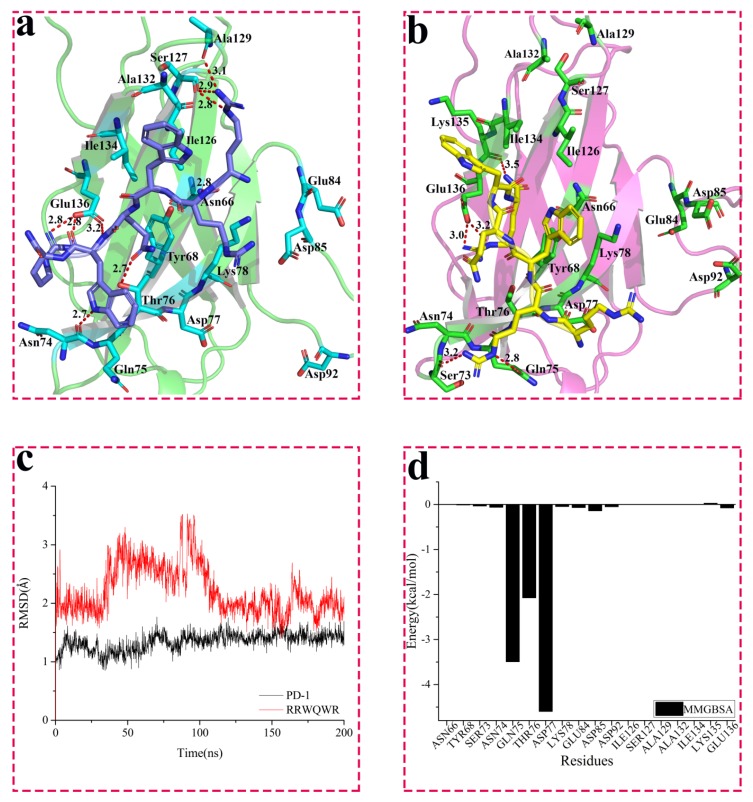
Interaction between WANG-005 (RRWQWR) and PD-1. (**a**) Interaction between WANG-005 (RRWQWR) and PD-1; (**b**) Interaction between WANG-005 (RRWQWR) and PD-1 after 200 ns MD simulation; The root mean square deviation (RMSD) for the backbone (@C, CA, N, O) of PD-1 (**c**) and Molecular Mechanics/Generalized Born Surface Area (MM/GBSA) (**d**) of PD-1 with RRWQWR for 200 ns molecular dynamics (MD) simulation.

The binding mode of PD-1 with WANG-005 (RRWQWR) was shown in Figure 5a. The results showed that the guanidyl of Arg1 on WANG-005 formed three hydrogen bonds with the carbonyl of Ser127 (2.8 and 2.9 Å) and Ala129 (3.1 Å) on PD-1. The acyl of Arg1 on WANG-005 also formed a strong hydrogen bond with the amino of Asn66 (2.8 Å). Similarly, strong hydrogen bonding (2.7 Å) between the hydroxyl of Thr76 on PD-1 and the carboxyl of Gln4 occurred on the peptide. The Gln4 on the peptide formed a hydrogen bond (3.2 Å) with the carbonyl of Glu136 on PD-1 as well. Interestingly, the hydroxyl of Glu136 formed two strong hydrogen bonds (2.8 Å) with the guanidyl of Arg6. In addition, the results indicated that the Trp5 on WANG-005 formed hydrogen interactions (2.7 Å) with the acyl of Gln75 on PD-1.

To affirm the interactions described previously and further investigate the dynamic interactions, a 200 ns MD simulation was carried out for the system of PD-1 complexed with WANG-005. More specifically, the RMSD of protein fluctuated slightly initialed on the 1.3-Å range until 85 ns, and then remained stable at 1.4 Å for the rest of simulation time (black line in Figure 5c). Moreover, the RMSD of WANG-005 dropped from ~3.5 to ~2.9 Å in 80 to 110 ns, and then fluctuated around this value. The average structure of the last 90 ns simulations was taken and is shown in Figure 5b. The guanidyl of Arg2 formed two hydrogen bonds with the carbonyl of Ser73 (3.2 Å) and Gln75 (2.8 Å) on PD-1. Two hydrogen bonds (3.0 and 3.2 Å) were also observed between the guanidyl of Arg6 and the carboxyl of Glu136 on PD-1. Additionally, the acyl of Glu136 on PD-1 formed a hydrogen bond (3.5 Å) with the acyl at the end of the peptide chain.

To evaluate the contribution of key residues to the binding between PD-1 and WANG-005, the MM/GBSA method was utilized to decompose free energy, and the analysis results are presented in Figure 5d and Table S3. The energy contribution of Gln75, Thr76, and Asp77 had a higher total energy in comparison to that of the other resides. These key residues worked together to promote the interaction of PD-1 and WANG-005.

#### 3.3.4. SPR-Based Binding Studies on Designed Peptides and PD-1

To verify the result of the *in-silico* docking and molecular dynamics simulation, SPR-based binding studies were conducted to measure the binding affinities between the synthetic peptide and the active extracellular domain of human PD-L1. Firstly, PD-L1 with a concentration gradient was run over the immobilized PD-L ectodomain to confirm the PD-1 function that interacts with PD-L1 (Figure S4 in the Supplementary Information). The binding data was processed by the kinetic model of the evaluation software, showing that the affinity of PD-1 and PD-L1 had a KD value of 0.8825 ± 0.0050 μM, which is comparable to previous reports [52].

Therefore, the affinity of synthetic peptide inhibitors with PD-1 was analyzed by the kinetic model via the flow of the immobilized protein. The binding affinity results between three peptides and PD-1 are shown in Table 2, Figure S4, and Table S4. According to the obtained preliminary data, all of the peptides could interact with PD-1. The most potent FRWWR-NH_2_ had a KD value of 1.6333 ± 0.3088 μM, which was stronger than that of KRWWR-NH_2_ and RRWQWR-NH_2_. The tests suggested the affinities of all peptides were weaker than but comparable to the binding level of PD-1 and PD-L1. Furthermore, FRWWR-NH_2_ had a moderate binding affinity at the PD-1 protein, which is similar to the reported D-peptide antagonists and caffeoylquinic acid compounds [19,52], indicating that the peptide designed in the present study had the potency to block the PD-1–PD-L1 interaction. Since the affinity of PD-1 for immobilized PD-L1 was measured with a KD of 0.01 to 0.05 μM and three of our peptides were shown to bind with KD values between 1 and 6 μM, the values of our peptides could be overestimated due to the experiment setup. To further validate our results, we also selected another two peptides (WANG-006 and WANG-007) with lower docking scores for testing and found that they did not show binding affinities, indicating our method/protocol was reasonable.

## 4. Conclusions

In the present work, we combined virtual screening, molecular docking, and MD simulation to explore the potential of our in-house peptide(s) as PD-1 inhibitor(s)/modulator(s). The development of our inhibiting peptide involved the characterization of binding through SPR technology. Our in-house peptides can act via, at least partially, the PD-1/PD-L1 pathway. The result from the present study demonstrated that WANG-003, WANG-004, and WANG-005 can bind to the PD-1 receptor with moderate affinity and may interrupt PD-1–PD-L1 interaction. Amino acid residues in PD-1 that were found to play an important role in the recognition of our peptide included Thr68, Glu75, Thr76, Asp85, Ile126, and Glu136. Some limitations of our in-house peptides should be recognized. As natural amino acid-containing peptides, our in-house peptides may have low stability in plasma serum due to degradation by proteolytic enzymes. Our peptide(s) may serve as a new chemical probe for further studies. For incident, non-natural amino acids are currently being considered for our next generation peptide. Moreover, we also found that when the peptides are short enough, they are more stable with fewer side effects. Finally, our peptides may be used as a foundation for the future design of low molecular weight peptides/small molecules that target PD-1. The binding pocket and key residue information can be used to virtually screen additional low molecular weight peptides and small molecules. As mentioned previously, we were only able to screen 30 peptides from our in-house library. For future research, we plan to screen additional virtual libraries to discover additional potent peptides with fewer side effects.

## Figures and Tables

**Table 1:** The detailed information on HPLC and MS of in-house peptides.

NO.	Name	Peptide/Protein	MW (g/mol)		HPLC
			Calculated	Observed	Purity
1	-	PD-L1	26000	-	-
2	WANG-003	KRWWR-NH_2_	831.00	830.00	95.96%
3	WANG-004	FRWWR-NH_2_	849.00	848.40	98.79%
4	WANG-005	RRWQWR-NH_2_	1045.23	1045.20	98.50%
5	WANG-006	YVAM-NH_2_	481.60	481.20	98.93%
6	WANG-007	YVAE-NH_2_	479.52	479.20	98.34%

**Table 2:** Affinity values of the interaction of peptides with PD-1.

No.	Name	Peptide	KD (μM)	Docking Score	Figure
1	PD-L1	-	0.8825 ± 0.0050	-	S4a
2	WANG-003	KRWWR-NH_2_	3.3527 ± 1.0276	9.36	S4b
3	WANG-004	FRWWR-NH_2_	1.6333 ± 0.3088	9.93	S4c
4	WANG-005	RRWQWR-NH_2_	5.1537 ± 2.9329	10.01	S4d
5	WANG-006	YVAM-NH_2_	NA	5.34	-
6	WANG-007	YVAE-NH_2_	NA	5.91	-

## Supplementary Materials

The following are available online: Figure S1: Characterization of WANG-003 by analytical HPLC chromatogram and MS Spectrum; Figure S2: Characterization of WANG-004 by analytical HPLC chromatogram and MS Spectrum; Figure S3: Characterization of WANG-005 by analytical HPLC chromatogram and MS Spectrum. Figure S4: Affinity between PD-1 and peptides (PD-L1/WANG-003/WANG-004/WANG-005). Table S1: Energy decomposition analysis (EDA) of complex KRWWR-PD-1 (WANG-003); Table S2: Energy decomposition analysis (EDA) of complex FRWWR-PD-1(WANG-004); Table S3: Energy decomposition analysis (EDA) of complex RRWQWR-PD-1(WANG-005); Table S4: Affinity values of the interaction of peptides with PD-1.
