# Supplementary material for: PD-1-Targeted Discovery of Peptide Inhibitors by Virtual Screening, Molecular Dynamics Simulation, and Surface Plasmon Resonance
Source: Molecules. 2019 Oct 21;24(20):3784. doi: 10.3390/molecules24203784 (PMC6833008; doi:10.3390/molecules24203784)
Supplement: Supplementary file 1 [file molecules-24-03784-s001.pdf]

Supplementary information

PD-1 targeted discovery of peptide inhibitors by virtual screening, molecular dynamics simulation, and surface plasmon resonance

Yuanqiang Wang [1,2,3,7,#,*], Haiqiong Guo [1,2,3#], Zhiwei Feng [4,5,#], Siyi Wang [4,5], Yuxuan Wang [1,2,3], Qingxiu He [1,2,3], Guangping Li [1,2,3], Weiwei Lin [4,5,], Xiang-Qun Xie [4,5,6,*] and Zhihua Lin [1,2,3*]

1 School of Pharmacy and Bioengineering, Chongqing University of Technology, Chongqing, 400054, China;

2 Chongqing Key Laboratory of Medicinal Chemistry and Molecular Pharmacology, Chongqing, 400054, China;

3 Chongqing Key Laboratory of Target Based Drug Screening and Effect Evaluation, Chongqing, 400054, China;

4 Department of Pharmaceutical Sciences and Computational Chemical Genomics Screening Center, School of Pharmacy, University of Pittsburgh, Pittsburgh, Pennsylvania 15261, United States;

5 National Center of Excellence for Computational Drug Abuse Research, Drug Discovery Institute, University of Pittsburgh, Pittsburgh, Pennsylvania 15261, United States;

6 Departments of Computational Biology and Structural Biology, School of Medicine, University of Pittsburgh, Pittsburgh, Pennsylvania 15261, United States;

7 State Key Laboratory of Silkworm Genome Biology, Southwest University, Chongqing, 400715, China.

* Correspondence: wangyqnn@cqut.edu.cn (Y.W.); xix15@pitt.edu (X-Q.X.); zhlin@cqut.edu.cn (Z.L.)

# These authors contributed equally to this work.

1.The characterization of synthetic novel tripeptides

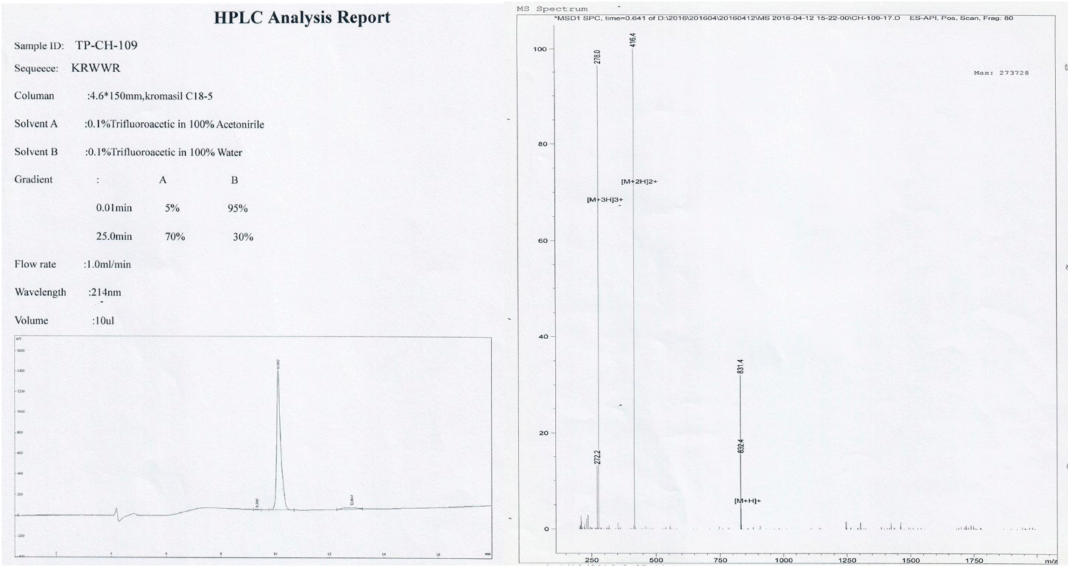

Figure S1. Characterization of WANG-003 by analytical HPLC chromatogram and MS Spectrum. HPLC conditions: a linear gradient of 100% acetonitrile (with 0.1% TFA) and 100% water (with 0.1% TFA) over 20 min on a kromasil C18-5 (4.6 × 250 mm) column, the total flow is 1.0ml/min. MS Spectrum observed 830.00 Da (calculated 831.00 Da, average isotopes).

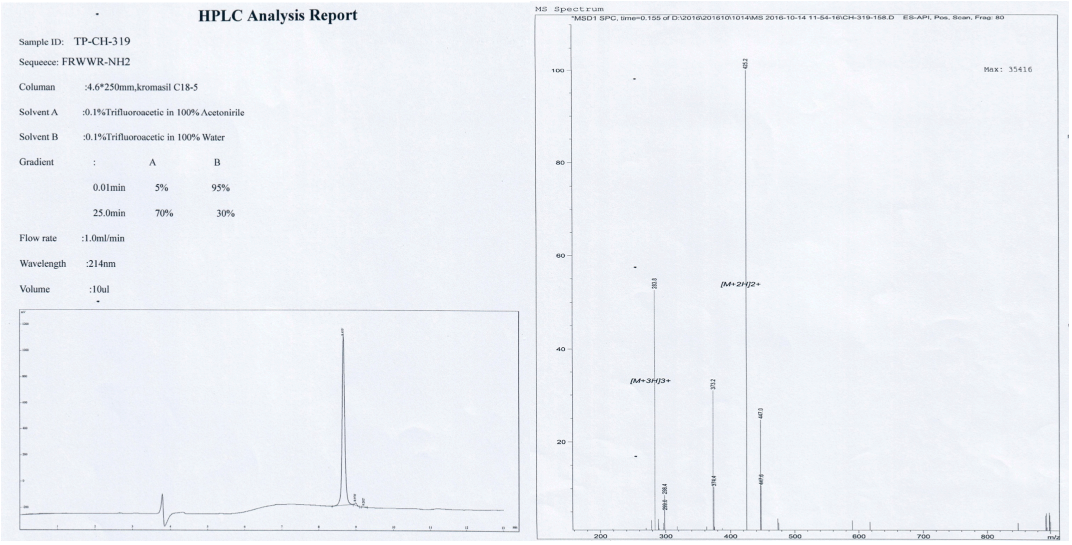

Figure S2. Characterization of WANG-004 by analytical HPLC chromatogram and MS Spectrum. HPLC conditions: a linear gradient of 100% acetonitrile (with 0.1% TFA) and 100% water (with 0.1% TFA) over 20 min on a kromasil C18-5 (4.6 × 250 mm) column, the total flow is 1.0ml/min. MS Spectrum observed 848.40 Da (calculated 849.00 Da, average isotopes).

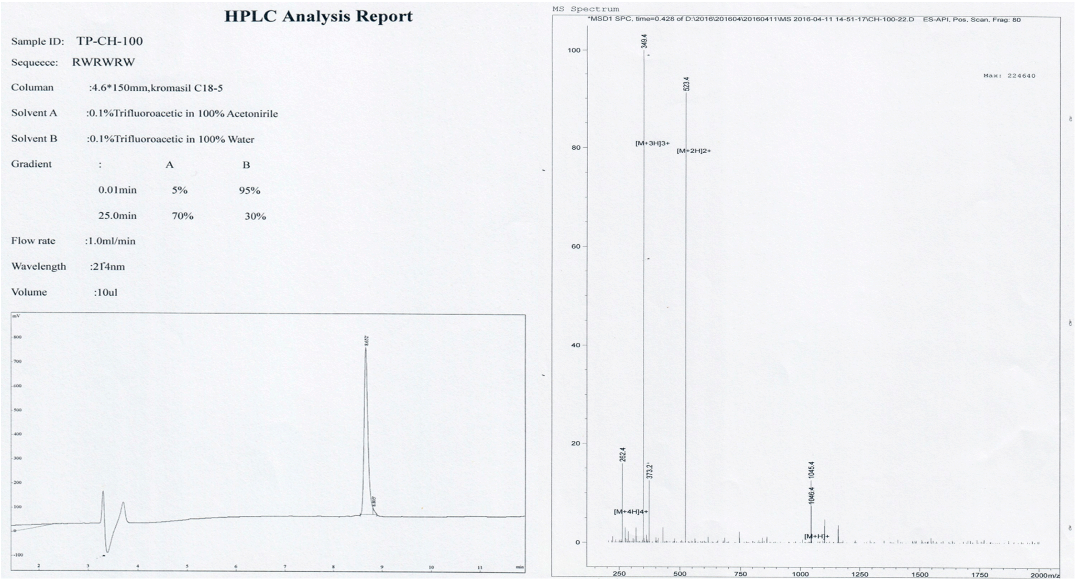

Figure S3. Characterization of WANG-005 by analytical HPLC chromatogram and MS Spectrum. HPLC conditions: a linear gradient of 100% acetonitrile (with 0.1% TFA) and 100% water (with 0.1% TFA) over 20 min on a kromasil C18-5 (4.6 × 250 mm) column, the total flow is 1.0ml/min. MS Spectrum observed 1045.20 Da (calculated 1045.23 Da, average isotopes).

2. Energy decomposition analysis (EDA) of complex Peptides-PD-1

Table S1 Energy decomposition analysis (EDA) of complex KRWWR-PD-1 (WANG-003)

Key residue	van de Waals	Electrostatic	Polar Solvation	non-polar Solvation	Total
Asn66	-0.0335	-0.0408	0.0798	0.0000	0.0055
Trp67	-0.0137	-1.6893	1.6139	0.0000	-0.0891
Tyr68	-0.0297	0.2599	-0.2632	0.0000	-0.0330
Thr76	-0.0082	-0.2593	0.2937	0.0000	0.0262
Asp77	-0.0375	-28.9362	28.6548	0.0000	-0.3188
Lys78	-0.4560	45.9752	-45.5838	-0.7032	-0.7677
Glu84	-0.0274	-27.9178	27.9209	-0.0001	-0.0244
Asp85	-0.0675	-70.9181	56.6490	-0.7085	-15.0452
Arg86	-0.4660	31.7826	-36.0521	-0.9230	-5.6585
Gln88	-0.7981	-10.6212	4.7068	-0.8201	-7.5326
Gln91	-0.1257	-8.3063	3.2540	-0.3562	-5.5342
Ile126	-0.0125	0.2448	-0.2418	-0.0002	-0.0097
Ser127	-0.0011	-0.6545	0.6493	0.0000	-0.0063
Ala129	-0.0001	-0.6270	0.6226	0.0000	-0.0045
Ala132	-0.0010	-0.2703	0.2684	0.0000	-0.0030
Ile134	-0.0044	0.4990	-0.4946	0.0000	0.0000
Glu136	-0.0021	-19.9261	19.8540	0.0000	-0.0742

Table S2 Energy decomposition analysis (EDA) of complex FRWWR-PD-1(WANG-004)

Key residue	van de Waals	Electrostatic	Polar Solvation	non-polar Solvation	Total
Asn66	-0.0062	-0.0749	0.0773	0.0000	-0.0038
Tyr68	-0.0041	-0.0166	0.0095	0.0000	-0.0112
Thr76	-0.0012	-0.1203	0.1194	0.0000	-0.0020

Asp77	-0.0012	-9.293	9.2630	0.0000	-0.0313
Lys78	-0.0115	15.0627	-14.9713	-0.0031	0.0767
Glu84	-0.0252	-15.9198	15.8004	-0.0036	-0.1481
Asp85	-0.0180	11.9910	-11.9319	-0.0043	0.0368
Ile126	-0.0479	0.3632	-0.3524	-0.0023	-0.0393
Ser127	-0.0348	-0.7992	0.8010	-0.0025	-0.0355
Leu128	-0.3875	-0.5746	0.5348	-0.3198	-0.7471
Ala129	-0.0516	-0.7036	0.6832	-0.0160	-0.0879
Pro130	-0.0446	-0.3093	0.3033	-0.0134	-0.0639
Ala132	-0.0202	0.3424	-0.3336	-0.0005	-0.0119
Ile134	-0.0124	0.2160	-0.2120	-0.0013	-0.0097
Glu136	-0.0012	-9.4807	9.4486	0.0000	-0.0333

Table S3 Energy decomposition analysis (EDA) of complex RRWQWR-PD-1(WANG-005)

Key residue	van de Waals	Electrostatic	Polar Solvation	non-polar Solvation	Total
Asn66	-0.0039	0.2123	-0.2073	-0.0039	0.0011
Tyr68	-0.0308	1.1271	-1.1058	-0.0026	-0.0121
Ser73	-0.0178	-0.8906	0.8728	-0.0001	-0.0356
Asn74	-0.0710	-0.9653	0.9823	-0.0104	-0.0643
Gln75	-0.4269	-3.1770	0.7262	-0.6145	-3.4923
Thr76	-0.3619	-3.6248	2.2426	-0.3298	-2.0740
Asp77	-0.4177	-47.6671	44.0944	-0.6076	-4.5979
Lys78	-0.1221	26.3230	-26.1915	-0.0557	-0.0464
Glu84	-0.0018	-19.2116	19.1440	0.0000	-0.0694
Asp85	-0.0079	-22.2908	22.1617	-0.0039	-0.1409
Asp92	-0.0010	-16.9067	16.8565	0.0000	-0.0512
Ile126	-0.0019	0.1500	-0.1489	0.0000	-0.0009

Ser127	-0.0001	-0.3864	0.3838	0.0000	-0.0026
Ala129	0.0000	-0.3665	0.3646	0.0000	-0.0019
Ala132	0.0000	-0.1889	0.1877	0.0000	-0.0012
Ile134	-0.0013	0.3563	-0.3537	0.0000	0.0013
Lys135	-0.0007	13.5721	-13.5454	0.0000	0.0260
Glu136	-0.0021	-20.1961	20.1216	0.0000	-0.0766

3.SPR measurement of hPD-1 and peptides to immobilized hPD-L1

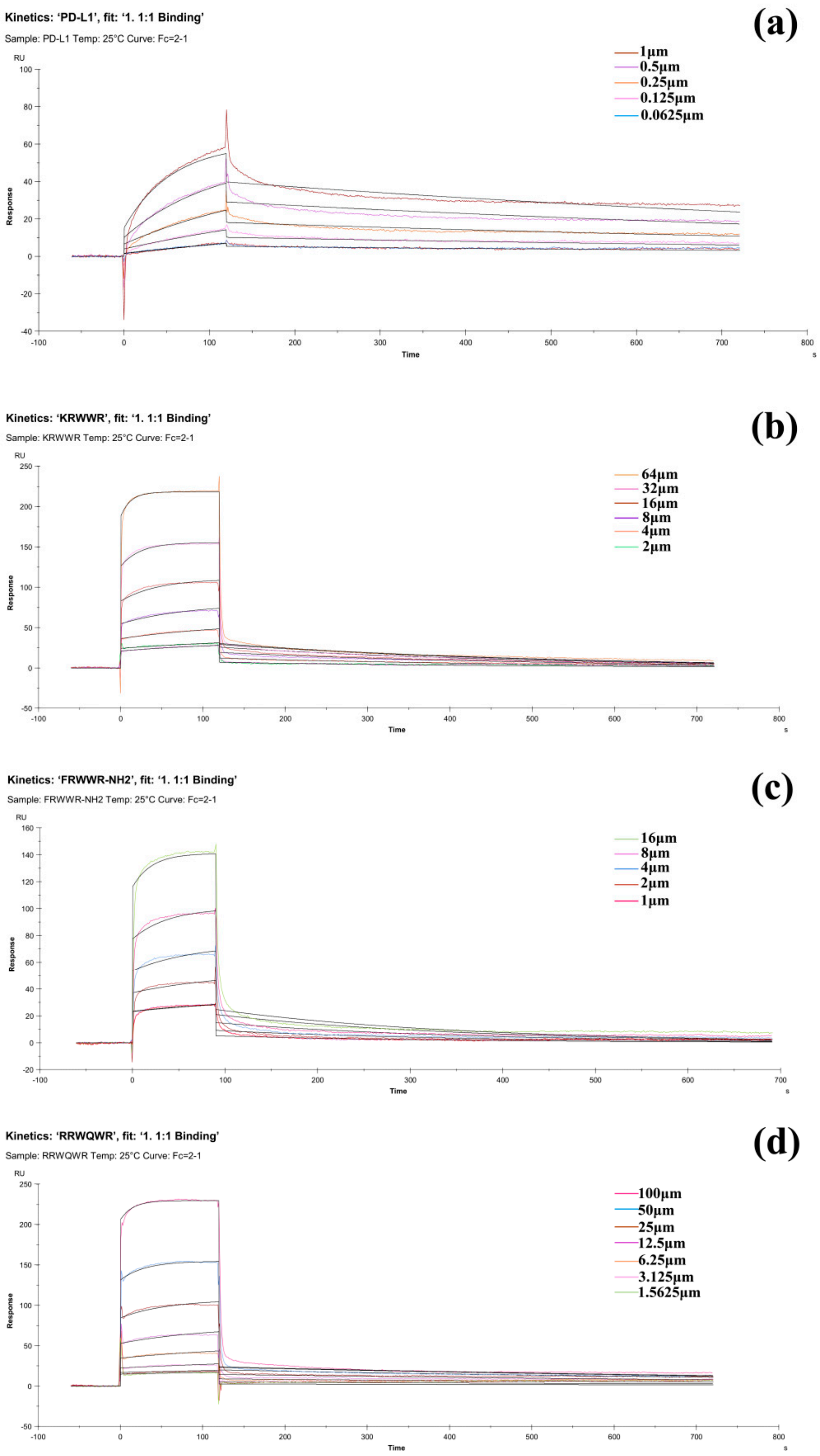

Figure S4. Affinity between PD-1 and peptides (PD-L1/WANG-003/WANG-004/WANG-005). A series of concentrations of peptides (PD-L1) were run over PD-1 to obtain the affinity between PD-1 and peptides (PD-L1) by kinetic analysis.

4. Affinity values of the interaction of peptides with PD-1

Table S4 **Affinity values of the interaction of peptides with PD-1**

No.	Name	Peptide	K_a(1/Ms)	K_d(1/s)	KD(μM)
			$(6.3450\pm0.6400)\times10^4$	$(5.5640\pm0.1700)\times10^{-2}$	0.8770
1	PD-L1	—	$(7.2750\pm0.6500)\times10^4$	$(6.4130\pm0.1700)\times10^{-2}$	0.8815
			$(7.5260\pm0.6100)\times10^4$	$(6.6920\pm0.1900)\times10^{-2}$	0.8892
			$(1.2340\pm0.0410)\times10^3$	$(2.5840\pm0.0800)\times10^{-3}$	2.0950
2	WANG-003	$KRWWR\text{-}NH_2$	$(0.9669\pm0.0590)\times10^3$	$(4.4590\pm0.2500)\times10^{-3}$	4.6120
			$(1.0890\pm0.0240)\times10^3$	$(3.6500\pm0.0470)\times10^{-3}$	3.3510
			$(2.7740\pm0.1100)\times10^3$	$(3.829\pm0.0860)\times10^{-3}$	1.3800
3	WANG-004	$FRWWR\text{-}NH_2$	$(2.1160\pm0.0960)\times10^3$	$(4.3770\pm0.1000)\times10^{-3}$	2.0680
			$(2.7650\pm0.1300)\times10^3$	$(4.0140\pm0.1400)\times10^{-3}$	1.4520
			$(0.6575\pm0.1500)\times10^3$	$(1.0280\pm0.0350)\times10^{-3}$	1.5640
4	WANG-005	$RRWQWR\text{-}NH_2$	$(0.4358\pm0.0290)\times10^3$	$(3.8120\pm0.2500)\times10^{-3}$	8.7480
			$(0.4938\pm0.0150)\times10^3$	$(2.5430\pm0.0520)\times10^{-3}$	5.1490
